# Mathematical model for predicting oxygen concentration in tilapia fish farms

**DOI:** 10.1038/s41598-021-03604-1

**Published:** 2021-12-16

**Authors:** El-Sayed Khater, Adel Bahnasawy, Hossam El-Ghobashy, Yousry Shaban, Faisal Elsheikh, Solaf Abd El-Reheem, Mohamed aboegela

**Affiliations:** 1 Agricultural and Biosystems Engineering Department, Faculty of Agriculture, Benha University, P.O. Box 13736, Moshtohor, Toukh, Kalubia Egypt; 2 Institute of Agricultural Engineering Research, Agriculture Research Center, Doki, Giza Egypt

**Keywords:** Ecology, Environmental sciences, Engineering

## Abstract

The main aim of this research is to develop a mathematical model to predict the dissolved oxygen in recirculating aquaculture system. The oxygen consumption of the model through the fish respiration and nitrification and the oxygen addition of the model through oxygen generator and water pumping. The effect of different water temperatures (24, 26, 28, 30 and 32 °C) on the dissolved oxygen consumption through fish respiration, biofilter and nitrification and fish growth were studied. An experiment to measure oxygen consumed by fish respiration and biofilteration and fish growth with the growth period and to validate the model results was carried out. The oxygen consumption predicted by the model was in a good agreement with those measured by the system. The oxygen consumption by fish respiration ranged 12.04 to 47.53 g O_2_ m^−3^ h^−1^ experimentally, while it was from 12.01 to 46.06 g O_2_ m^−3^ h^−1^ theoretically. The predicted and measured oxygen consumption through biofilteration values ranged from 0.43 to 21.91 and 0.45 to 23.09 g O_2_ m^−3^ h^−1^, respectively. The individual fish weight from the system ranged from 3.00 to 209.52 g experimentally while it was from 3.00 to 226.25 g theoretically during the whole period.

## Introduction

Dissolved Oxygen (DO) has a very important parameter in water quality determination in fish farming, where low levels of DO affect the palatability of feed which reduce the feeding which in turn affect the growth. Low DO in ponds is related to measuring carbon dioxide (CO_2_) and unionized ammonia (NH_3_) which are toxic to fish. DO concentrations less than 5 mg L^−1^ affect the growth deeply. Fish can no longer survival at 2 mg L^−1^ dissolved oxygen^1^.

A Recirculating Aquaculture System (RAS) is defined as an aquaculture system that incorporates which reuse the water by 10% after treatment. The concept of RAS is to reuse a volume of water through continual treatment and delivery to organisms being cultured. To treat the water in RAS it has to have a system can handle high amount of water to be able to produce higher quantities of fish economically. Generally, this system needs different types of filters, pumps and tanks to improve the quality of water which in turn controlling disease and ensure higher production^2^.

In RAS, aeration is very important to control DO by either adding pure oxygen or mixed air with high oxygen. Controlling dissolved oxygen by aeration is very important in high loading capacity of fish such as tilapia or catfish. However, commercial recirculating aquaculture system has to have an aerator instead of using liquid oxygen, where aerating water with low dissolved oxygen to saturation point but using liquid oxygen could reach supersaturation^3^.

One of the limiting factors in recirculating aquaculture system is the oxygen concentration in water, where, it dissolves in water poorly. The saturation values for growing cold-water species is 10.08 mg L^−1^ at 15 °C and 8.26 mg L^−1^ at 25 °C for warm water farming force to replenish constantly oxygen content in water to ensure an efficient fish growth. The oxygen concentration threshold for warm water species is 50% (absolute value 4.13 mg L^−1^ at 25 °C) and 60% (6.48 mg L^−1^ at 15 °C) for salmonids at a fish growing tank effluent^4^.

The most critical water quality parameter is level of dissolved oxygen (DO) available to organisms in an aquaculture system is because it is essential to the metabolism of the majority of cultured fish and crustaceans^5^. Fish oxygen consumption rates depend on various factors including: environmental activity level DO concentration, water temperature, fish size, and time after feeding^6^. Reduced oxygen levels cause lethal and sub-lethal effects including: reduced feeding and growth rates, lower food conversion efficiencies and higher susceptibility to disease in various aquatic organisms^7^.

Many researchers have studied the oxygen mass transfer simulation. They found good agreement between simulation and experimental results. They concluded that the equilibrium DO level decreased with increasing temperature and that the oxygen transfer efficiency is higher for smaller values of gas flow rate. They found also, that the aeration system to aquaculture resulted in a decrease in the equilibrium DO level due to the oxygen consumption by the fish^1,4,8,9^.

Stocking densities, feed addition, temperature and the tolerance of the fish species to hypoxia affect the oxygen requirements of a system. Low dissolved oxygen levels may quickly result in high stress in fish, nitrifying biofilter malfunction and indeed significant fish losses. Accurate dissolved oxygen measuring and predicting are very important for fish production. The prediction of dissolved oxygen in recirculating aquaculture system one of the most important way to determine of fish production and essential to the design and evaluate of this system, therefore, the main aim of this study is to develop a mathematical model to predict the dissolved oxygen consumption at different water temperature and fish weight.

## Model development

### Dissolved oxygen model

**Figure 1:**
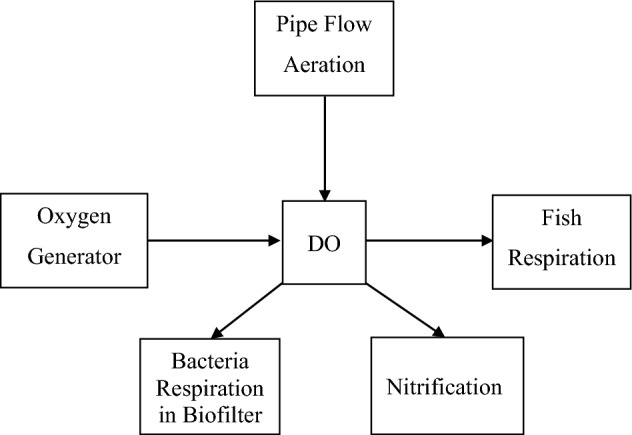
Dissolved oxygen model.

The dissolved oxygen in this model had a number of interactions to consider. Oxygen consumption through the processes of both respiration and nitrification. On the other hand, the water receives oxygen through water agitation as it is pumped through the system and from the oxygen generator. Oxygen is added to the water by oxygen generator and flow aeration (Fig. 1).

The required oxygen supplementation is a sum of the pervious components as follows:

1 $$ DO_{FR} + DO_{B} + DO_{N} = DO_{\sup } + DO_{PF} $$

where DO_FR_ is the dissolved oxygen consumption through fish respiration, g O_2_ m^−3^ h^−1^. DO_B_ is the dissolved oxygen consumption through the biofilter, g O_2_ m^−3^ h^−1^. DO_N_ is the dissolved oxygen consumption through nitrification, g O_2_ m^−3^ h^−1^. DO_PF_ is the dissolved oxygen addition through pipe flow, g O_2_ m^−3^ h^−1^. DO_sup_ is the required oxygen supplementation (oxygen generator), g O_2_ m^−3^ h^−1^.

The rate of change in DO concentration in fish tank:

2 $$ \frac{dDO}{{dt}} = DO_{FR} + DO_{B} + DO_{N} - DO_{PF} $$

where $\frac{dDO}{{dt}}$ is the rate of change in DO concentration during the time interval, g O_2_ m^−3^ h^−1^. dt is the rate of change in the time interval, h

After calculating oxygen concentration for each element at each time step, the net oxygen change is then added to or subtracted from the previous time step`s oxygen concentration. DO concentrations can be calculated at any time (t) as:

3 $$ DO_{t} = DO_{t - 1} + \left( {\frac{dDO}{{dt}} \cdot dt} \right) $$

where DO_t_ is the DO concentration (g m^−3^) at time t. DO_t−1_ is the DO concentration (g m^−3^) at time t−1.

The rate of oxygen consumption through fish respiration can be calculated on water temperature and average fish weight. This calculation is shown in the following equation^10^:

4 $$ FR = 2014.45 + 2.75W - 165.2T + 0.007W^{2} + 3.93T^{2} - 0.21WT $$

5 $$ DO_{FR} = \frac{FR \times SD}{{1000}} $$

where FR is rate of oxygen consumption through fish respiration, mg O_2_ kg^−1^ fish. h^−1^. W is average of individual fish mass, g. T is water temperature, °C. SD is the stocking density of fish, kg m^−3^.

The correlation coefficient for the equation was 0.99. Data used in preparing the equation ranged from 20 to 200 g for fish weight and from 24 to 32 °C.

The rate of oxygen consumption through nitrification is calculated in terms of Total Ammonia Nitrogen (TAN) that is converted from ammonia to nitrate. The rate found in the literature is 4.57 g O_2_ g^−1^ TAN^6^.

The oxygen consumption in nitrification process can be calculated as^11^:

6 $$ DO_{N} = 4.57 \times K_{NR} \times {{{\text{Nr}}} \mathord{\left/ {\vphantom {{{\text{Nr}}} {\text{V}}}} \right. \kern-\nulldelimiterspace} {\text{V}}} $$

7 $$ K_{NR} = 0.1\left( {1.08} \right)^{{\left( {T - 20} \right)}} $$

8 $$ Nr = \frac{{0.03 \times F_{r} \times W \times N_{F} }}{24 \times 1000} $$

where K_NR_ is the coefficient of nitrification. N_r_ is the nitrification rate, g TAN h^−1^. F_r_ is the feeding ratio, % of body fish day^−1^. N_F_ is the number of fish. V is the water volume, m^3^.

The feeding ratio can be calculated as the following equation:

9 $$ F_{r} = 17.02 \times e^{{\left[ {{\raise0.7ex\hbox{${\left( {\ln W + 1.14} \right)^{2} }$} \!\mathord{\left/ {\vphantom {{\left( {\ln W + 1.14} \right)^{2} } { - 19.52}}}\right.\kern-\nulldelimiterspace} \!\lower0.7ex\hbox{${ - 19.52}$}}} \right]}} $$

The bacteria in the biofilter are a second source of oxygen consumption. Lawson explains that the biofilter oxygen demand is approximated 2.3 times the BOD_5_ production rate of fish^6^. The oxygen consumption of the biofilter is calculated using following equation:

10 $$ DO_{B} = \frac{{(2.3)\left( {BOD_{5} } \right)\left( {W_{n} } \right)}}{{\left( V \right)\left( {24} \right)\left( {1000} \right)}} $$

where BOD_5_ is average unfiltered BOD_5_ excretion rate, 2160 mg O_2_ kg^−1^ fish day^−1^. W_n_ is biomass, kg fish.

The water pumping cycle was a source of oxygen addition to the system. The amount of oxygen addition through the water pumping cycle was calculated on an hourly basis. The method of calculating aeration from a pipe is detailed by^12^:

11 $$ DO_{PF} = \frac{PC \times f \times E \times OTR}{V} $$

where PC is pump cycle length, h. f is pumping frequency, h^−1^. E is efficiency, %. OTR is oxygen transfer rate, g O_2_ h^−1^.

This model sums the DO_FR_, DO_B_, DO_N_, and DO_PF_ to determine the supplemental DO demand in kg h^−1^. This number can be used to estimate the oxygen consumption if pure oxygen transfers system is used.

### Fish growth model

Fish growth is affected by environmental and physical factors, such as water temperature, dissolved oxygen, unionized ammonia, photoperiod, fish stocking density, food availability, and food quality.

In order to calculate the fish growth rate (g day^−1^) for individual fish, the following model was used^13^ as it includes the main environmental factors influencing fish growth. These factors are temperature, dissolved oxygen and unionized ammonia.

12 $$ FGR = \left( {0.2919 \, \tau \, \kappa \, \delta \, \varphi \, h \, f \, W^{m} } \right) - K.W^{n} $$

Where FGR is the fish growth rate, g day^−1^. τ is the temperature factor (0 > τ < 1, dimensionless). к is the photoperiod factor (0 > к < 1, dimensionless). δ is the dissolved oxygen factor (0 > δ < 1, dimensionless). φ is the unionized ammonia factor (0 > φ < 1, dimensionless). h is the coefficient of food consumption (g^1-m^ day^−1^). ƒ is the relative feeding level (0 > ƒ < 1, dimensionless). K is the coefficient of catabolism.h, m, n are constants.

Water temperature affects the food intake^14^. Caulton^15^ described the relationship between temperature and feed intake for tilapias. Food intake rate reaches the maximum value when the temperature is in an optimal range. If the temperature is outside the optimal range, the food intake rate decreases. Food intake stops when the temperature is the limit range. The temperature factor (from 0 to 1) can be described as^16,17^.

13 $$ \tau = EXP\left\{ { - 4.6\left[ {\frac{{T_{opti} - T}}{{T_{opti} - T_{\max } }}} \right]^{4} } \right\}\quad {\text{if}}\;\;{\text{T}} \prec {\text{T}}_{{{\text{opti}}}} \, $$

14 $$ \tau = EXP\left\{ { - 4.6\left[ {\frac{{T - T_{opti} }}{{T_{\max } - T_{opti} }}} \right]^{4} } \right\}\quad {\text{if}}\;\;{\text{T}} \ge {\text{T}}_{{{\text{opti}}}} $$

where T_min_ is the below this temperature fish stop eating, °C. T_max_ is the above this temperature fish stop eating, °C. T_opti_ is the optimum temperature for fish taking food, °C.

The catabolism term is also affected by temperature. The effect is described as^18^:

15 $$ K = K_{\min } {\text{ exp}}\left[ {{\text{s}}\left( {{\text{T}} - {\text{T}}_{{{\text{min}}}} } \right)} \right] $$

where K_min_ is the coefficient of fasting catabolism at T_min_, g^1−n^ h^−1^. s is a constant.

The effect of DO on fish growth is described in three stages. When DO is below the minimum limits level, DO_min_ fish feeding stops. When DO is above a critical level, DO_crit_, DO has no effect on feeding. When DO is between DO_min_ and DO_crit_ feeding is affected by DO^18^.

16 $$ \delta = 1.0\quad {\text{if}}\;\;{\text{DO}} \succ {\text{DO}}_{{{\text{crit}}}} $$

17 $$ \delta = \frac{{DO - DO_{\min } }}{{DO_{crit} - DO_{\min } }}\quad {\text{if}}\;\;{\text{DO}}_{{{\text{min}}}} \le DO \le DO_{crit} $$

18 $$ \delta = 0.0\quad {\text{if}}\;\;{\text{DO}} \prec {\text{DO}}_{{{\text{crit}}}} $$

Unionized ammonia, NH_3_, is toxic to fish^19^. The effects of unionized ammonia can be simulated using an equation similar to that for DO^18^. When NH_3_ is higher than NH_3max_, then the fish stop feeding. When NH_3_ is lower than the critical value, NH_3crit_, then there is no effect on feeding. When the concentration of NH_3_ is higher than the critical value, NH_3crit_ and lower than a maximum value, NH_3max_, then food intake will decrease as the concentration of NH_3_ increases. The function can be decreased as^18^.

19 $$ \varphi = 1.0\quad {\text{if}}\;\;{\text{NH}}_{{3}} \prec NH_{{{\text{3crit}}}} $$

20 $$ \varphi = \frac{{NH_{3\max } - NH_{3} }}{{NH_{3\max } - NH_{3crit} }}\quad {\text{if}}\;\;{\text{NH}}_{{{\text{3crit}}}} \le NH_{3} \le NH_{3\max } $$

21 $$ \varphi = 0.0\quad {\text{if}}\;\;{\text{NH}}_{{3}} \succ NH_{{{\text{3crit}}}} $$

Caulton^20^ indicates that many cultured fish species including tilapias tended to feed only during daylight hours. Photoperiod factor (к), based on 12:12 h of light–dark cycle and used for adjusting daily food consumption, is expressed as follow:

22 $$ \kappa = {\text{photoperiod/12}} $$

where, photoperiod is the day time between sunrise and sunset (h), which can be estimated from sunrise and sunset hour angle calculations^21^. The constant of 12 is the photoperiod in the 12:12 h of light dark cycle.

The fish growth rate is dependent on the amount of food and the quality of available. To determine the value of the relative feeding level “ƒ” to be used in our case, we used the model at progressive values of “ƒ” starting from zero, step 0.01 up to 1.0 and compare the results with those obtained by^22^.

Equation is used to calculate the accumulate growth starting by one gram of individual fish to the marketable weight of 250 g.

23 $$ {\text{W}}_{{\text{n}}} = W_{n - 1} + FGR $$

24 $$ {\text{Amount}}\;{\text{of}}\;{\text{feeding}}\;{\text{(kg/day)}} = F_{r} \times W_{n} \times {\text{No}}.\;{\text{of}}\;{\text{fish/100,000}} $$

where n is the number of day from the start

**Figure 2:**
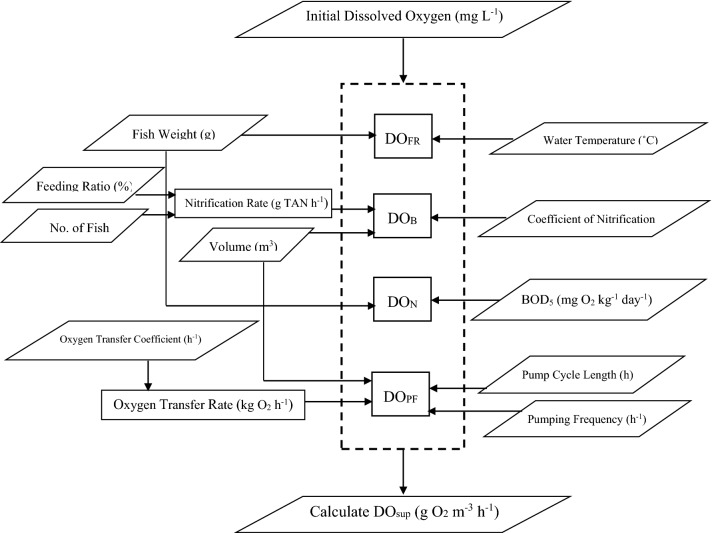
Flowchart of the model.

All computational procedures of the model were carried out using Excel spreadsheet. The computer program was devoted to mass balance for predicting the dissolved oxygen consumed through aquacultural recirculating system. Figure 2 shows the flowchart of the model. The parameters used in the model that were obtained from the literature are listed in Table 1.

**Table 1:** The parameters used in the mass balance.

Parameter	Unit	Value	Source
BOD_5_	mg O_2_ kg^−1^ fish day^−1^	2160	^6^
PC	h	1	
F	h^−1^	1	
E	%	50	^12^
OTR	g O_2_ h^−1^	36.8	^12^
C	–	0.236	^17^
H	–	0.81	^17^
M	–	0.67	^17^
N	–	0.81	^18^
T_min_	°C	15	^18^
T_max_	°C	40	^18^
T_opti_	°C	28	^18^
K_min_	–	0.25	^18^
S	–	0.015	^18^
DO_crit_	mg L^−1^	5	^13^
DO_min_	mg L^−1^	3	^13^
NH_3max_	mg L^−1^	0.6	^13^
NH_3crit_	mg L^−1^	0.025	^23^

## Experimental procedures

The main experiment was carried out at intensive fish farm, Faculty of Agriculture Moshtohor, Benha University, Egypt (latitude 30° 21′ N and 31° 13′ E). During the 2020/2021 season to validate the model results. All experimental protocols of this study were approved by the research committee in the Faculty of Agriculture Moshtohor, Benha University.

### System description

**Figure 3:**
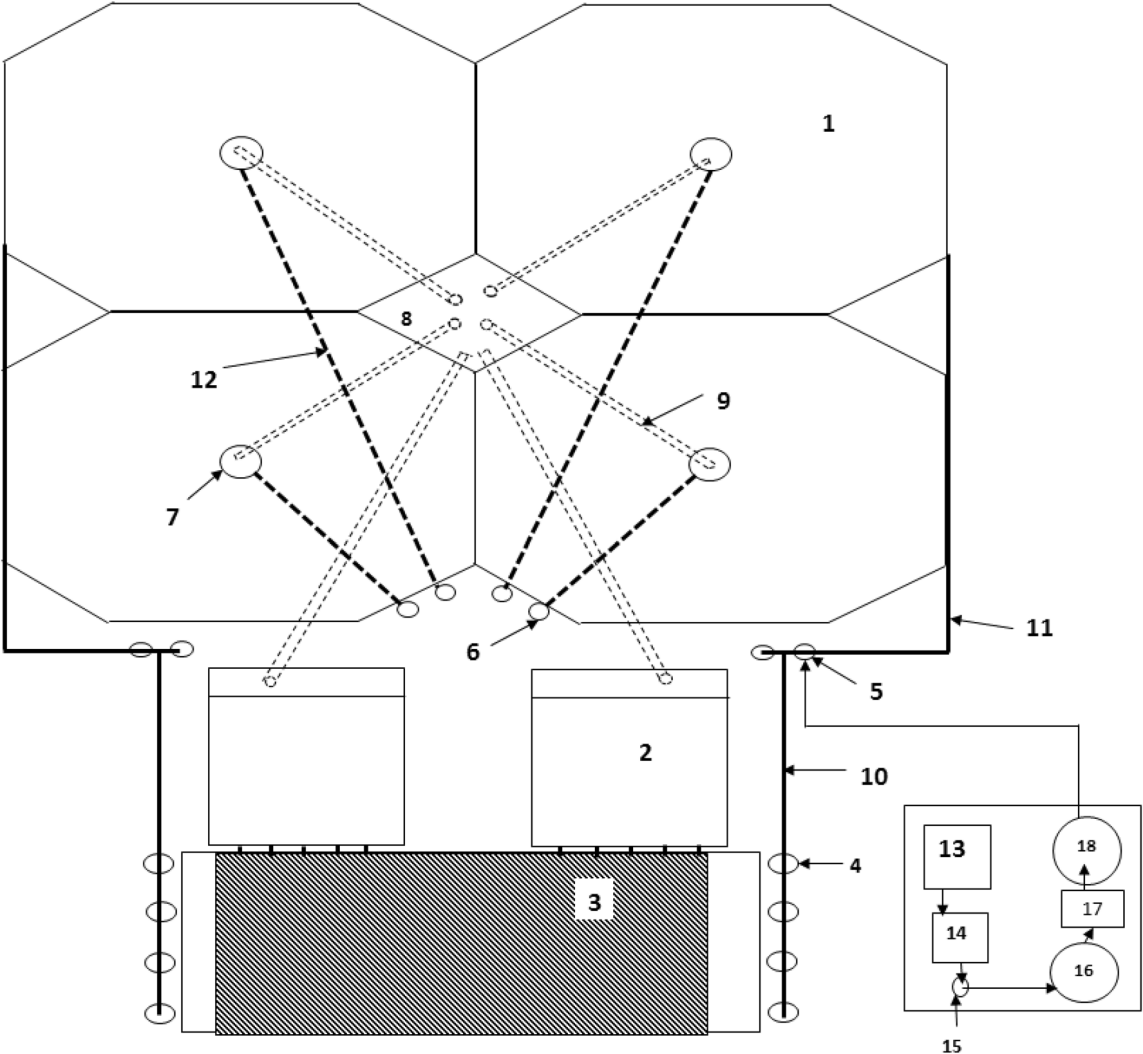
Intensive fish farm. (1) Fish tank (2) Screen filter (3) biological filter (4) Pump (5) Oxygen mixer (6) Hydrocyclone (7) Particle Trap (8) Collecting tank (9) PVC pipe φ 250 mm (10) PVC pipe φ 110 mm (11) PVC pipe φ 90 mm (12) PVC pipe φ 110 mm (13) Compressor (14) Refrigeration unit (15) Filtration unit (16) Air tank (17) Oxygen generator (18) Oxygen tank

Figure 3 illustrates the experimental setup. It shows the intensive fish farm (recirculating aquaculture system) which consists of fish tanks, hydrocyclone, screen filter, biological filter, oxygen generator and oxygen mixer.

Four fish tanks are an octagonal in shape and made from concrete has to openings for both settleable and suspended solids. The water volume used in each tank is 150 m^3^ and has a height of 2.0 m. Each tank is provided with a particle trap in the center for water drain waste solids. The first opening allows for 1–15% of the total flow, the second opening allows for 85–99% of the total flow.

The hydrocyclone is used to remove the settleable solids which made from stainless steel and has inlet diameter of 50 mm, overflow diameter of 50 mm, height of 1000 mm, top diameter of 350 mm, underflow of 50 mm and cone angle of 68°.

Two drum screen filters used in this system which has dimensions of 1.20 m in diameter and 2.0 m long. The filter was made from stainless steel at private company for steel industry. The fine mesh silk 60 micron was used a media of screening. The filter was driven by one motor of 1.0 kW power and 1500 rpm and a gearbox was used to reduce the rotation speed 500 times to give the recommended rotating speed (3 rpm).

Trickling biological filter used in this system, has 8.0 m in long, 4.0 m width and 4.0 m high. The filter was made from concrete. Used plastic sheets were used as a media. The total volume of media used in this system is 96 m^3^.

Pure oxygen used in this system source of oxygen gas was oxygen generator. Adding pure oxygen gas to water by oxygen mixer. The water and oxygen enter the top of the oxygen mixer, as the water and oxygen move downward. Oxygen generator is used to provide the oxygenation system with its requirements of pure oxygen- It is consists of air compressor (Model BOGE – Flow rate 15 m^3^ h^−1^ – Head 10 bar – Power 25 kW, Germany), Refrigeration unit, Filtration unit, 1 m^3^ stainless steel tank for storage air, oxygen generator (Model BOGE – Flow rate 10.75 m^3^ h^−1^ – Head 6.25 bar – power 1 kW, Germany) and 1 m^3^ stainless steel tank for storage oxygen pure.

The water was circulated by four pumps (Model PEDROLLO – Flow Rate 30 m^3^ h^−1^ – Head 48 m – Power 4.0 kW, Italy) from the biological filter tank to the fish tanks. Different pipes were used to provide tanks with solution in a closed system.

### Methods

Tilapia nilotica fingerlings (20,000 fingerlings for each tank with an individual weight of 3 g), which were used in the beginning of experiment, were brought from the World Fish Center (WFC), Abbassa, Abou-Hammad Sharkia, Egypt. The fish was weighed every ten days and the flow rate was adjusted according to the growth rate. The daily feed rates at different fish sizes were applied according to^22^ as shown in Table 2 and the feed pellet diameter was prepared according to^24^ as shown in Table 3. Feeding was stopped during weighing process.

**Table 2:** The recommended feeding rates for different size of tilapia in tanks and estimated growth rates at 28 °C.

Weight (g)		Growth rate (g/day)	Growth period (day)	Feeding rate (%) of fish mass
Initial	Final			
0.02	0.5–1	–	30	15–20
0.5–1	5	–	30	10–15
5	20	0.5	30	7–10
20	50	1.0	30	4–7
50	100	1.5	30	3.5–4
100	250	2.5	50	1.5–3.5
250	450	3.0	70	1.0–1.5

**Table 3:** The recommended pellet size for tilapia.

Fish size (g)	Pellet diameter (mm)
Fry: first 24 h	Liquefy
Fry: 2nd–10th day	0.5
Fry: 10th–30th day	0.5–1.0
1–30	1–2
20–120	2
100–250	2
> 250	4

#### Dissolved oxygen

Dissolved oxygen was recorded using a DO Meter (Model HANNA HI5421; Range: 0 to 90 mg L^−1^ ± 1.5%, Italy) hourly.

#### Calculations

Oxygen consumption was calculated based on the differences between the dissolved oxygen at inlet and outlet of the fish tank by the following formula:

25 $$ {\text{OC}} = \frac{{\left( {{\text{DO}}_{{{\text{in}}}} - DO_{out} } \right) \times Q}}{{W_{n} }} \, \times {1000} $$

where OC is the oxygen consumption, mg O_2_ kg^−1^ fish h^−1^. DO_in_ is the dissolved oxygen at inlet the fish tank, mg L^−1^. DO_out_ is the dissolved oxygen at outlet the fish tank, mg L^−1^. Q is the flow rate, m^3^ h^−1^.

All methods used in this study was carried out according to the guidelines regulations of Benha University. This study was carried out in compliance with the ARRIVE guidelines.

## Results and discussion

### Model experimentations

#### Oxygen consumption through fish respiration

**Figure 4:**
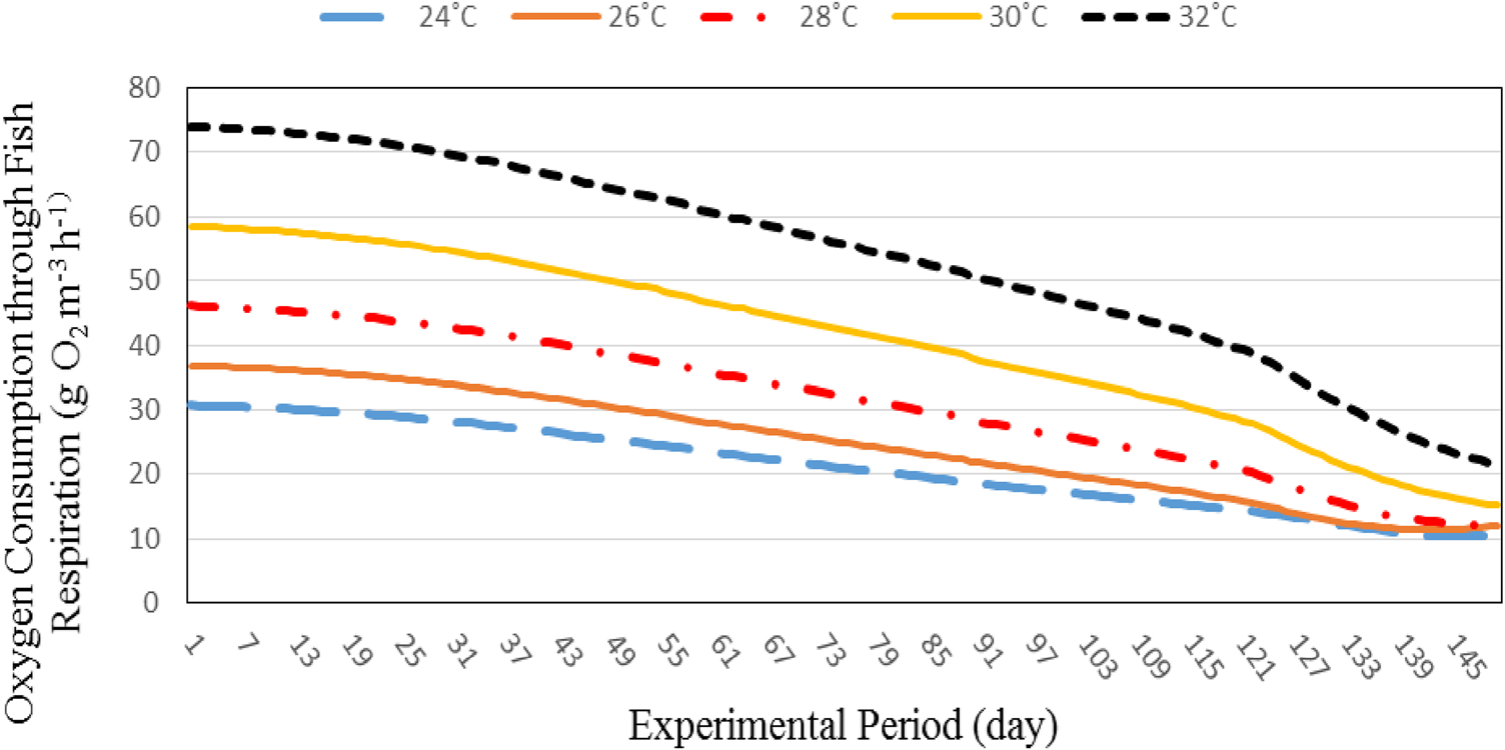
The effect of water temperature and individual fish weight on oxygen consumption through fish respiration.

Figure 4 shows the oxygen consumption through fish respiration at different water temperatures (24, 26, 28, 30 and 32 °C). The results indicate that the oxygen consumption through fish respiration increases with increasing water temperature. It could be that, when the water temperature increased from 24 to 32 °C the oxygen consumption through fish respiration increased from 30.65 to 74.05 g O_2_ m^−3^ h^−1^, respectively, at the same individual fish weight (3.00 g). At 226.25 g individual fish weight, the oxygen consumption through fish respiration increased from 10.42 to 21.57 g O_2_ m^−3^ h^−1^, when the water temperature increased from 24 to 32 °C, respectively. These results agreed with those obtained by^25^ who found that the higher oxygen consumption by fish due to high water temperature.

On the other hand, the results show the oxygen consumption through fish respiration decreases with increasing fish weight. It could be that, the oxygen consumption through fish respiration decreased from 30.65 to 10.42, 26.79 to 11.83, 46.06 to 12.01, 58.49 to 15.14 and 74.05 to 21.42 g O_2_ m^−3^ h^−1^ at 24, 26, 28, 30 and 32 °C, respectively, when the fish weight increased from 3.00 to 226.25 g. These results agreed with those obtained by^4^ who found the oxygen consumption rate of fish respiration on a per unit mass basis decreases as fish weight increases.

#### Oxygen consumption through nitrification

**Figure 5:**
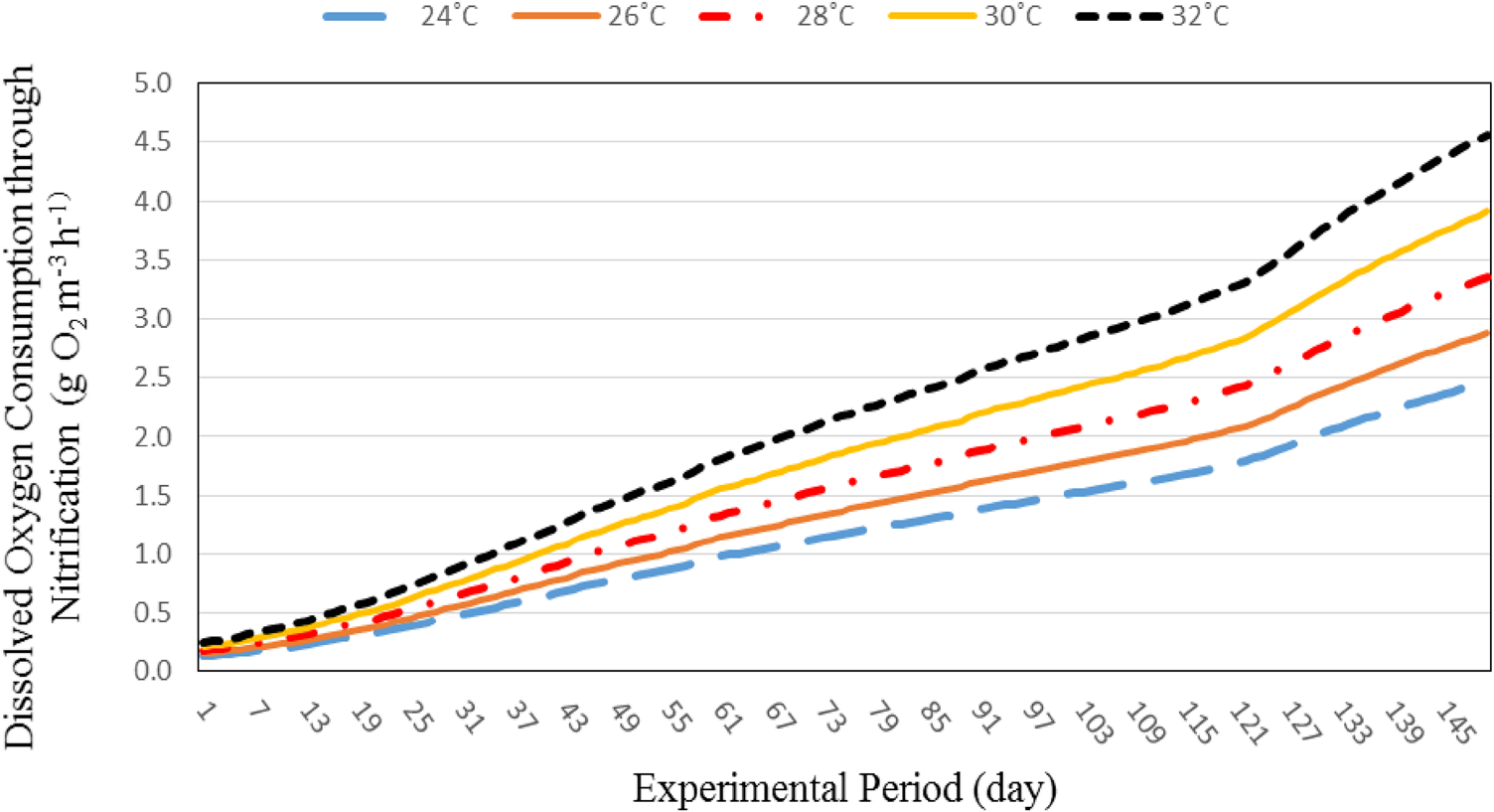
The effect of water temperature and individual fish weight on oxygen consumption through nitrification.

Figure 5 shows the oxygen consumption through nitrification at different water temperatures (24, 26, 28, 30 and 32 °C). The results indicate that the oxygen consumption through nitrification increases with increasing water temperature. It could be that, when the water temperature increased from 24 to 32 °C the oxygen consumption through nitrification increased from 0.13 to 0.24 and 2.46 to 4.56 g O_2_ m^−3^ h^−1^ at 3.00 and 226.25 g individual fish weight, respectively.

The results also indicate that, the oxygen consumption through nitrification increases with increasing fish weight. It could be that, the oxygen consumption through nitrification increased from 0.13 to 2.46, 0.16 to 2.87, 0.18 to 3.35, 0.21 to 3.91 and 0.24 to 4.56 g O_2_ m^−3^ h^−1^ at 24, 26, 28, 30 and 32 °C, respectively, when the fish weight increased from 3.00 to 226.25 g.

#### Total oxygen consumption through recirculating aquaculture system

**Figure 6:**
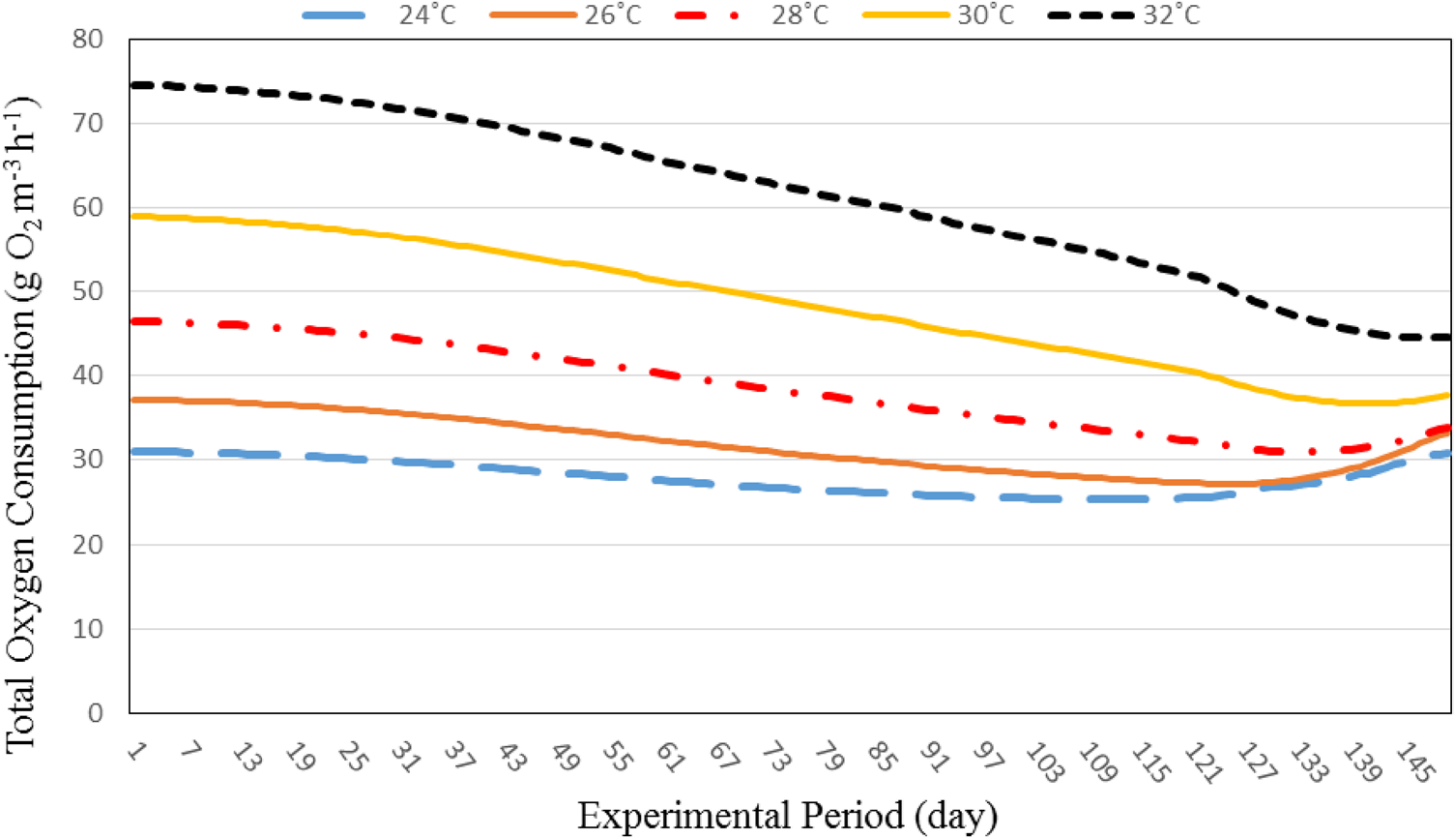
The effect of water temperature and individual fish weight on total oxygen consumption through recirculating aquaculture system.

Figure 6 shows the total oxygen consumption through recirculating aquaculture system (fish respiration, nitrification and biofilter) at different water temperatures (24, 26, 28, 30 and 32 °C). The results indicate that the total oxygen consumption through recirculating aquaculture system increases with increasing water temperature. It could be that, when the water temperature increased from 24 to 32 °C the oxygen consumption through recirculating aquaculture system increased from 31.04 to 74.55 g O_2_ m^−3^ h^−1^, respectively, at the same individual fish weight (3.00 g). At 226.25 g individual fish weight, the oxygen consumption through recirculating aquaculture system increased from 30.78 to 44.54 g O_2_ m^−3^ h^−1^, when the water temperature increased from 24 to 32 °C, respectively.

On the other hand, the results show the oxygen consumption through recirculating aquaculture system decreases with increasing fish weight. It could be that, the oxygen consumption through recirculating aquaculture system decreased from 31.04 to 30.78, 37.19 to 33.27, 46.49 to 33.92, 58.95 to 37.61 and 74.55 to 44.54 g O_2_ m^−3^ h^−1^ at 24, 26, 28, 30 and 32 °C, respectively, when the fish weight increased from 3.00 to 226.25 g.

#### Oxygen required for recirculating aquaculture system

**Figure 7:**
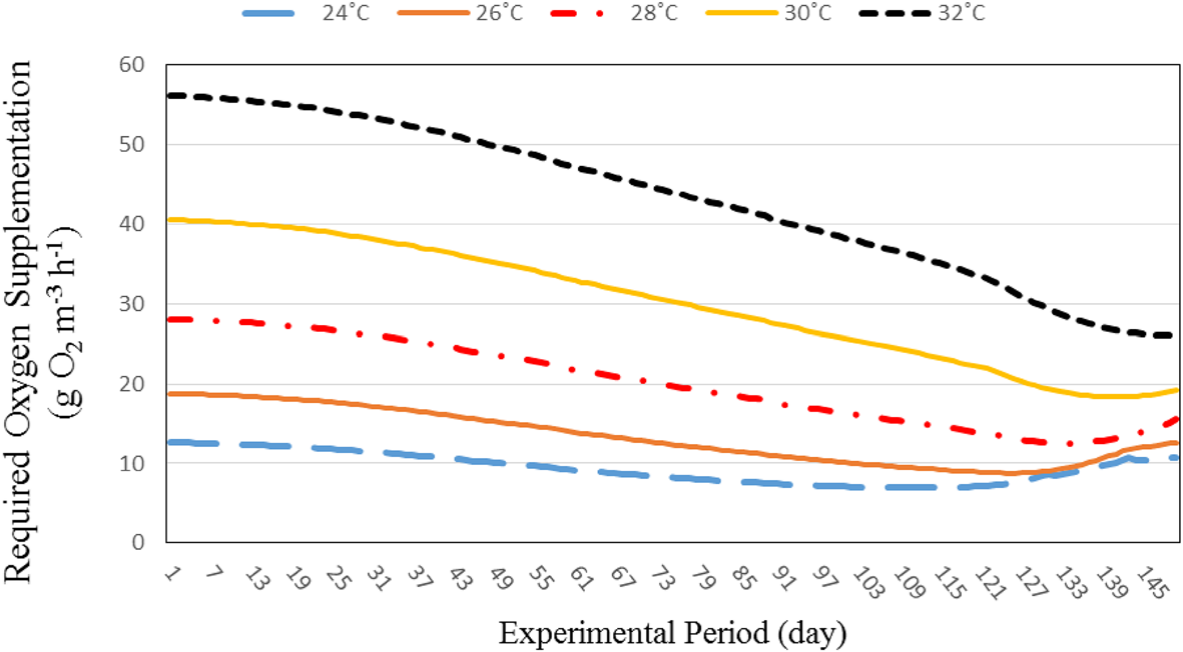
The effect of water temperature and individual fish weight on oxygen required for recirculating aquaculture system.

Figure 7 shows the oxygen required for recirculating aquaculture system at different water temperatures (24, 26, 28, 30 and 32 °C). The results indicate that the oxygen required for recirculating aquaculture system increases with increasing water temperature. It could be that, when the water temperature increased from 24 to 32 °C the oxygen required for recirculating aquaculture system increased from 12.63 to 56.15 g O_2_ m^−3^ h^−1^, respectively, at the same individual fish weight (3.00 g). Also, the oxygen required from recirculating aquaculture system increased from 10.74 to 26.14 g O_2_ m^−3^ h^−1^, when the water temperature increased from 24 to 32 °C, respectively at 226.25 g individual fish weight.

On the other hand, the results show the oxygen required for recirculating aquaculture system decreases with increasing fish weight. It could be that, the oxygen required for recirculating aquaculture system decreased from 12.65 to 10.74, 18.79 to 12.65, 28.09 to 15.52, 40.35 to 19.21 and 56.15 to 26.14 g O_2_ m^−3^ h^−1^ at 24, 26, 28, 30 and 32 °C, respectively, when the fish weight increased from 3.00 to 226.25 g.

#### Fish weight

**Figure 8:**
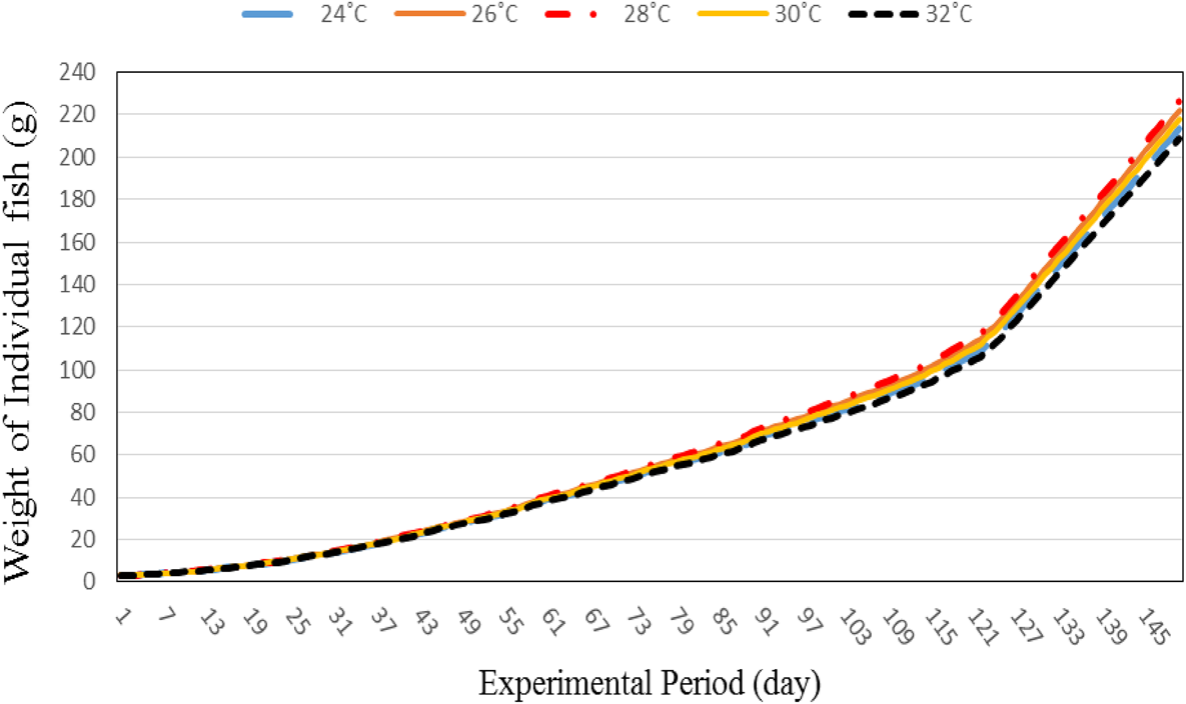
Individual fish weight at different water temperatures.

Figure 8 shows the predicated individual fish weight at different water temperatures (24, 26, 28, 30 and 32 °C). The results indicate that the fish weight increases with increasing experimental period. It could be seen that, the individual fish weight increased from 3.00 to 213.35, 3.00 to 222.02, 3.00 to 226.25, 3.00 to 217.78 and 3.00 to 209.92 g when the experimental period increases from 1 to 150 day, respectively. These results agreed with those obtained by^26^ who found that the fish weight increases with increasing growth period, the individual fish weight increased from 5.57 to 243.00 g when the growth period increases from 1 to 180 day, respectively.

The results also indicate that the highest value of individual fish weight (226.25 g) was found for 28 °C water temperature, while, the lowest value of individual fish weight (209.92 g) was found for 32 °C water temperature. Temperature is a critical factor that influences growth during the growth period, the growth rate for fish reared at 24 °C water temperature showed similar the growth rate for fish reared at 32 °C water temperature. The best fish growth rate was found at the optimum temperature. These results agreed with those obtained by^27^ who studied the effect of different water temperature (23, 26, 29 and 32 °C) on the fish growth and who found the highest value of fish growth was obtained at 29 °C water temperature.

### Model validation

#### Oxygen consumption through fish respiration

**Figure 9:**
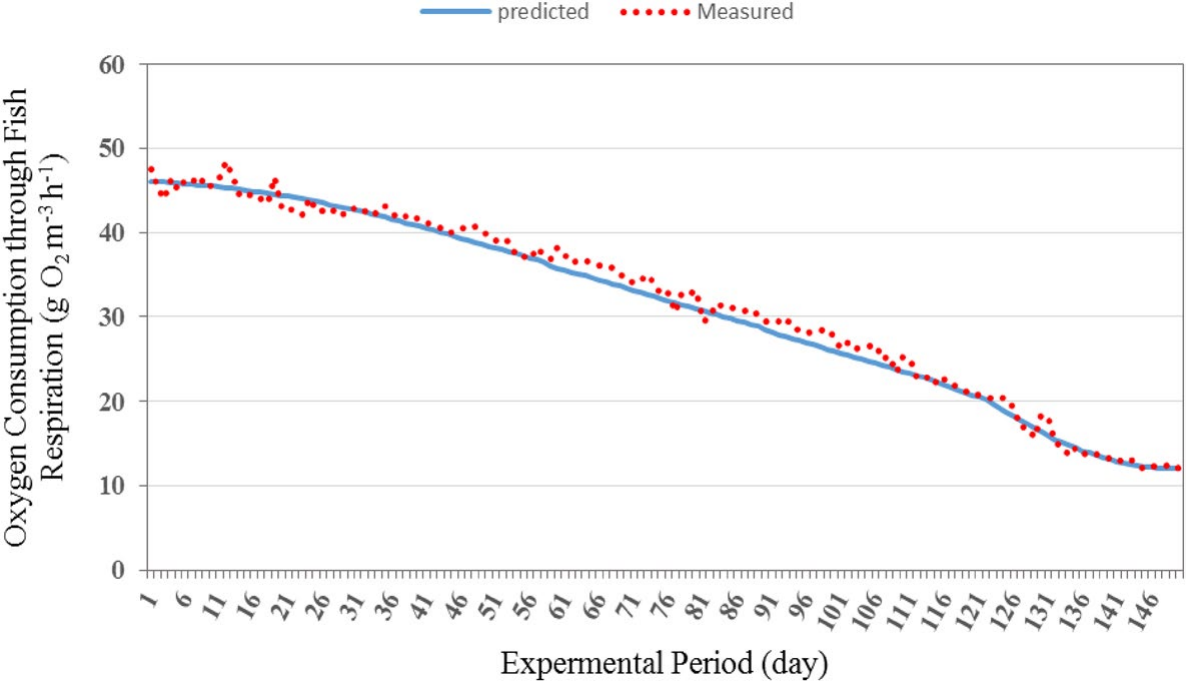
The predicted and the measured oxygen consumption of fish respiration during the whole period of fish growth under the recirculating aquaculture system.

**Figure 10:**
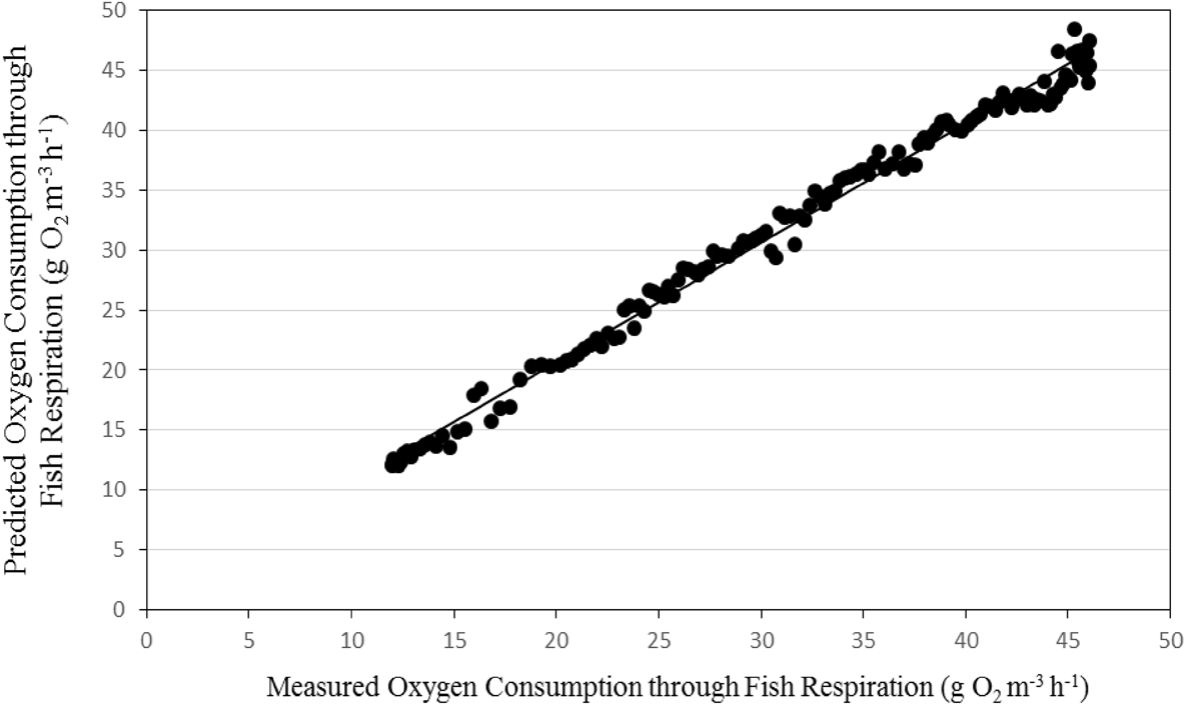
The comparison between the predicted and the measured oxygen consumption by fish respiration.

Figures 9 and 10 show the comparison between the predicted and the measured dissolved oxygen consumption of fish respiration for the recirculating aquaculture system (RAS). It could be seen that the predicted oxygen consumption of fish respiration values were between 12.01 and 46.06 g O_2_ m^−3^ h^−1^ and the measured oxygen consumption of fish respiration values are from 12.04 to 47.53 g O_2_ m^−3^ h^−1^ during the whole period. The predicted oxygen consumption of fish respiration showed a similar pattern to that of the measured oxygen consumption, but the predicted values were much lower.

The best fit for the relationship between the predicted and the measured values of oxygen consumption through fish respiration with coefficient of determination of 0.991 was in the following form with an error of 1.94%:

26 $$ OC_{{{\text{FRP}}}} = 0.994{\text{O}}C_{{{\text{FRM}}}} + 0.807\quad {\text{R}}^{{2}} = {0}{\text{.991}} $$

where OC_FRP_ is the predicted oxygen consumption of fish respiration, g O_2_ m^−3^ h^−1^. OC_FRM_ is the measured oxygen consumption of fish respiration, g O_2_ m^−3^ h^−1^.

The oxygen consumption rate of fish respiration on a per unit mass basis decreases as fish weight increases^4,28^. This was also evident in this study. Therefore, an increase in system biomass does not mean that there is an equal increase in feeding rate. In fact, suggested feeding rate (% body weight per day) decreases as fish size increases and as water temperature decreases.

#### Oxygen consumption through biofilteration

**Figure 11:**
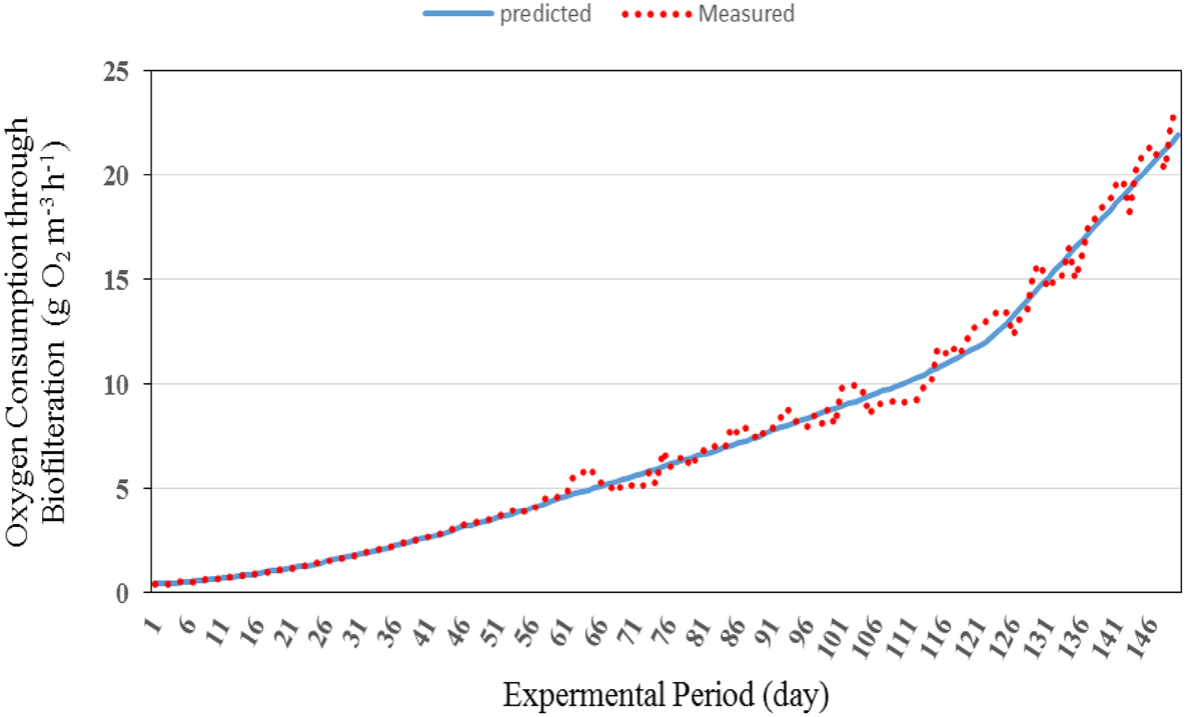
The predicted and the measured oxygen consumption of biofilteration during the whole period of fish growth under the recirculating aquaculture system.

**Figure 12:**
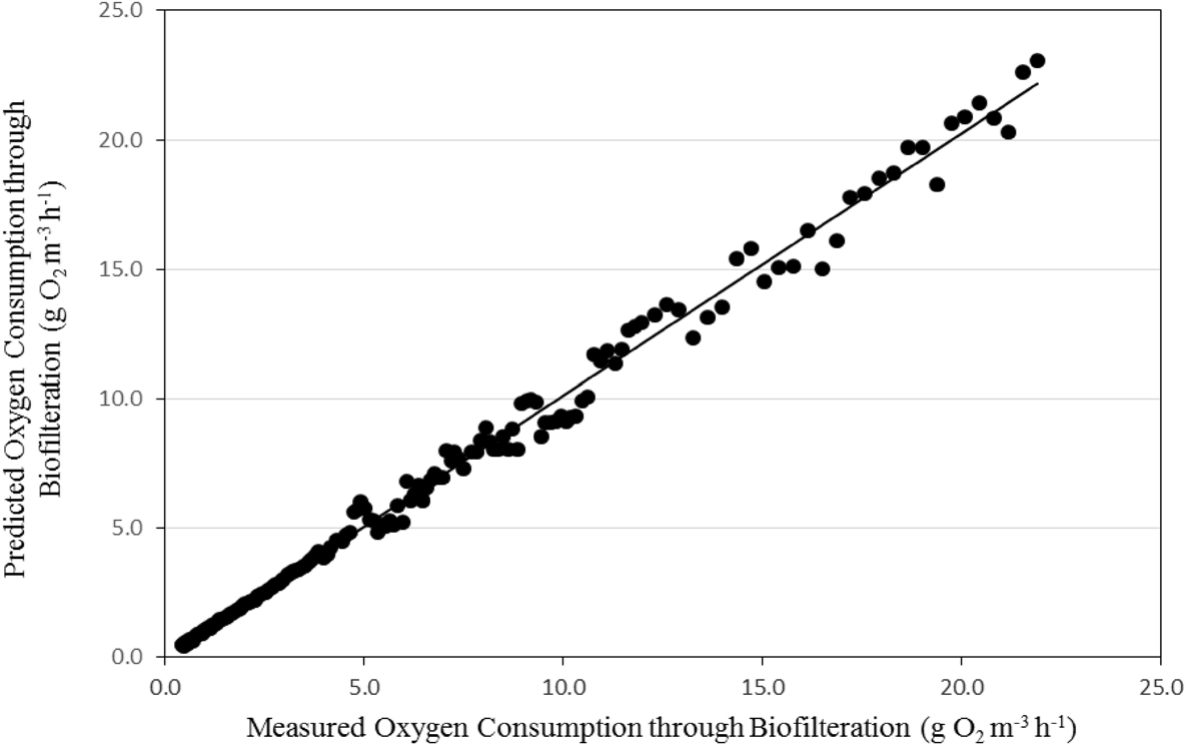
The comparison between the predicted and the measured oxygen consumption by biofilteration.

Figures 11 and 12 show the comparison between the predicted and the measured dissolved oxygen consumption by biofilteration for the recirculating aquaculture system (RAS). It could be seen that the oxygen consumption through biofilteration increased gradually. The results indicate also that, the average daily of oxygen consumption through biofilteration by the model was in a reasonable agreement with those measured, where, the oxygen consumption by biofilteration ranged from 0.43 to 21.91 g O_2_ m^−3^ h^−1^ theoretically while it was from 0.45 to 23.09 g O_2_ m^−3^ h^−1^ experimentally during the whole period.

The best fit for the relationship between the predicted and the measured values of oxygen consumption through biofilteration with coefficient of determination of 0.992 was in the following form with an error of 1.53%:

27 $$ OC_{{{\text{BFP}}}} = 1.013{\text{O}}C_{{{\text{BFM}}}} - 0.015\quad {\text{R}}^{{2}} = {0}{\text{.992}} $$

where OC_BFP_ is the predicted oxygen consumption of biofilteration, g O_2_ m^−3^ h^−1^. OC_BFM_ is the measured oxygen consumption of biofilteration, g O_2_ m^−3^ h^−1^.

#### Total oxygen consumption through the recirculating aquaculture system

**Figure 13:**
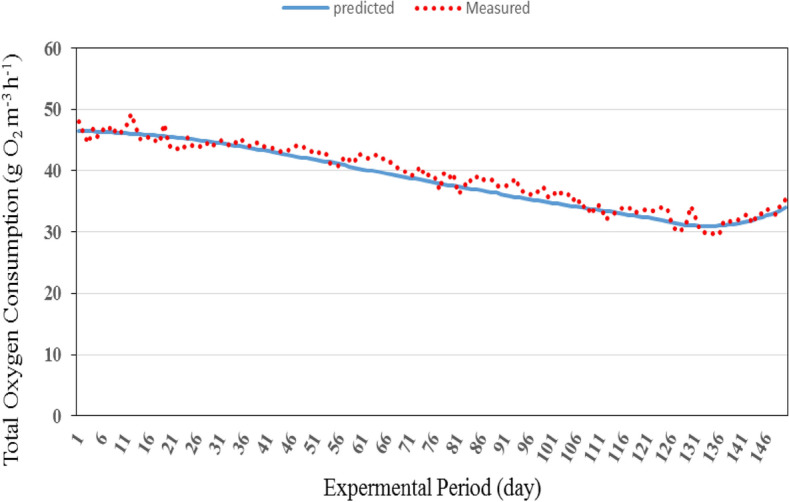
The predicted and the measured the total oxygen consumption through the recirculating aquaculture system during the whole period of fish growth.

**Figure 14:**
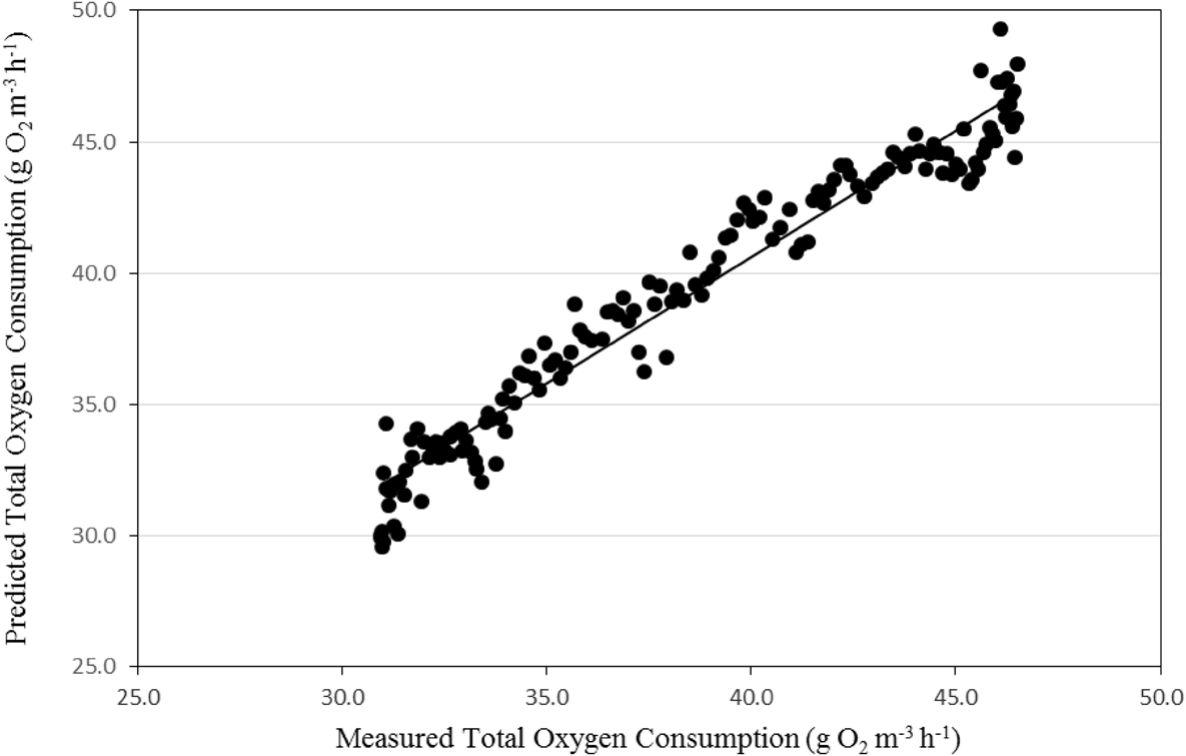
The comparison between the predicted and the measured total oxygen consumption through the recirculating aquaculture system.

Figures 13 and 14 show the comparison between the predicted and the measured total oxygen consumption for the recirculating aquaculture system (RAS). It could be seen that the total oxygen consumption through the recirculating aquaculture system decreased gradually and then increased to reach the peak after 133 day. The results indicate also that, the average daily of the total oxygen consumption through the system by the model was in a reasonable agreement with those measured, where, the total oxygen consumption through the recirculating aquaculture system ranged from 33.92 to 46.49 g O_2_ m^−3^ h^−1^ theoretically while it was from 35.22 to 47.98 g O_2_ m^−3^ h^−1^ experimentally during the whole period.

The best fit for the relationship between the predicted and the measured values of the total oxygen consumption through the recirculating aquaculture system with coefficient of determination of 0.954 was in the following form with an error of 1.61%:

28 $$ OC_{{{\text{SP}}}} = 0.964{\text{O}}C_{{{\text{SM}}}} + {2}{\text{.071}}\quad {\text{R}}^{{2}} = {0}{\text{.954}} $$

where OC_SP_ is the predicted total oxygen consumption through the system, g O_2_ m^−3^ h^−1^. OC_SM_ is the measured total oxygen consumption through the system, g O_2_ m^−3^ h^−1^.

#### Fish weight

**Figure 15:**
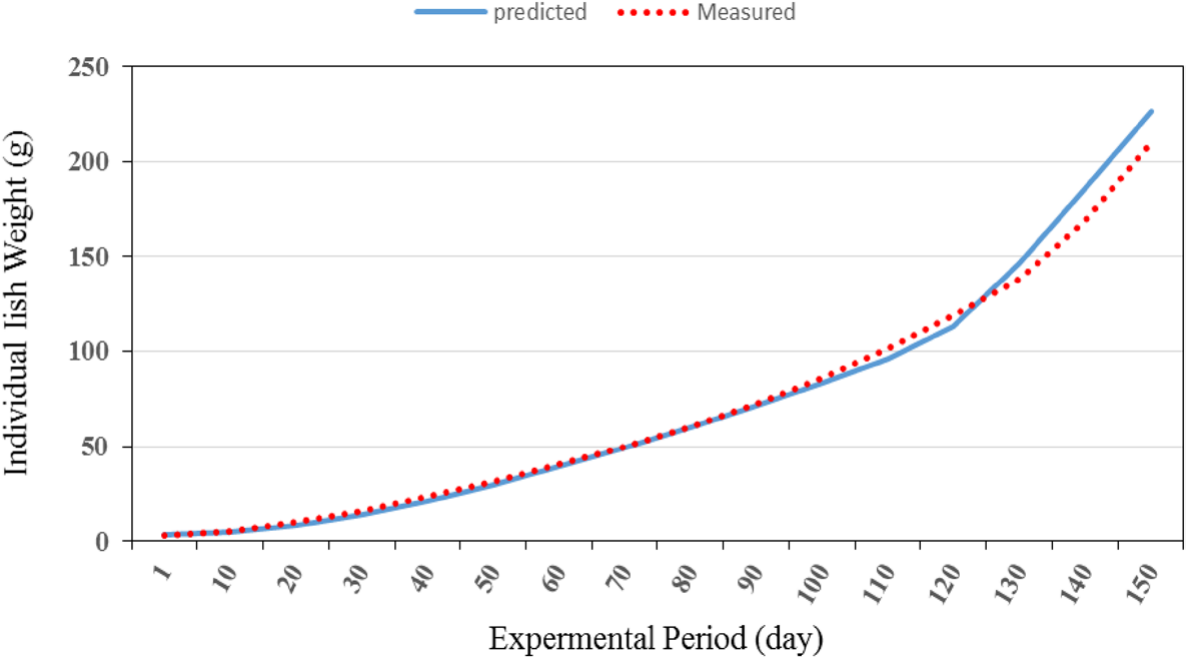
The predicted and the measured the individual fish weight during the whole period.

**Figure 16:**
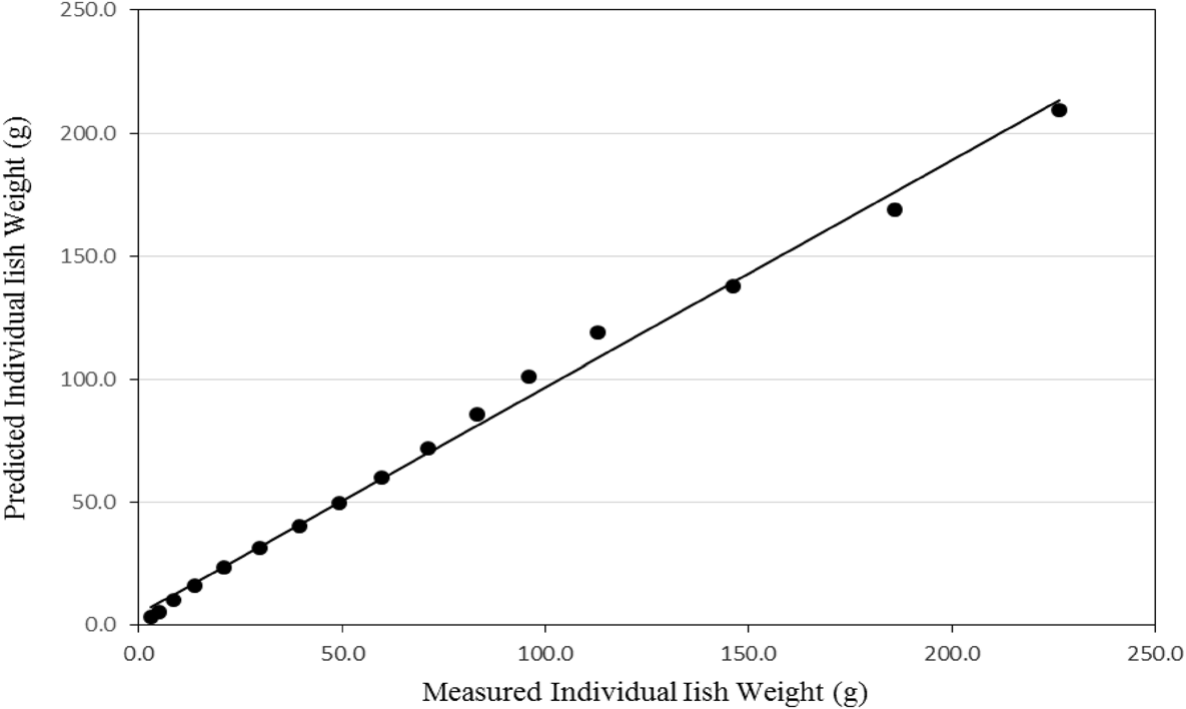
The comparison between the predicted and the measured total oxygen consumption through the recirculating aquaculture system.

Figures 15 and 16 show the comparison between the predicted and the measured individual fish weight from the recirculating aquaculture system during the whole period of fish growth. It could be seen that the individual fish weight from the system ranged from 3.00 to 209.52 g experimentally while it was from 3.00 to 226.25 g theoretically during the whole period. These results are in agreement with^29^.

The best fit for the relationship between the predicted and the measured values of individual fish weight from the recirculating aquaculture system during the whole period of fish growth with coefficient of determination of 0.993 was in the Eq. (29) with an error of 1.39%. These results agreed with those obtained by^26,30^ who found that the predicted fish weight obtained from the model is in good agreement with the measurements.

29 $$ FW_{{\text{P}}} = 0.923FW_{{\text{M}}} + {4}.384\quad {\text{R}}^{{2}} = {0}{\text{.993}} $$

where FW_P_ is the predicted individual fish weight, g. FW_M_ is the measured individual fish weight, g.

## Conclusions

A mathematical model was designed to predict the dissolved oxygen in recirculating aquaculture system. Study the effect of different water temperatures (24, 26, 28, 30 and 32 °C) on the dissolved oxygen consumption through fish respiration, biofilter and nitrification and fish growth. An experiment to validate the model results was carried out. The model was able to predict the dissolved oxygen at different water temperature and fish weight successively. The model results were in a good agreement with the measured ones.
